# Molecular characterization of *Plasmodium falciparum* uracil-DNA glycosylase and its potential as a new anti-malarial drug target

**DOI:** 10.1186/1475-2875-13-149

**Published:** 2014-04-17

**Authors:** Thidarat Suksangpleng, Ubolsree Leartsakulpanich, Saengduen Moonsom, Saranya Siribal, Usa Boonyuen, George E Wright, Porntip Chavalitshewinkoon-Petmitr

**Affiliations:** 1 Department of Protozoology, Faculty of Tropical Medicine, Mahidol University, Bangkok 10400, Thailand; 2 National Center for Genetic Engineering and Biotechnology, National Science and Technology Development Agency, Pathumthani 12120, Thailand; 3 Faculty of Medical Technology, Western University, Kanchanaburi 71170, Thailand; 4 Department of Molecular Tropical Medicine and Genetics, Faculty of Tropical Medicine, Mahidol University, Bangkok 10400, Thailand; 5 GLSynthesis Inc., One Innovation Drive, Worcester, MA 01605, USA

**Keywords:** *Plasmodium falciparum*, Malaria, Uracil-DNA glycosylase, Antimalarials, Drug target

## Abstract

**Background:**

Based on resistance of currently used anti-malarials, a new anti-malarial drug target against *Plasmodium falciparum* is urgently needed. Damaged DNA cannot be transcribed without prior DNA repair; therefore, uracil-DNA glycosylase, playing an important role in base excision repair, may act as a candidate for a new anti-malarial drug target.

**Methods:**

Initially, the native PfUDG from parasite crude extract was partially purified using two columns, and the glycosylase activity was monitored. The existence of malarial UDG activity prompted the recombinant expression of PfUDG for further characterization. The *PfUDG* from chloroquine and pyrimethamine resistant *P. falciparum* strain K1 was amplified, cloned into the expression vector, and expressed in *Escherichia coli*. The recombinant PfUDG was analysed by SDS-PAGE and identified by LC-MS/MS. The three dimensional structure was modelled. Biochemical properties were characterized. Inhibitory effects of 12 uracil-derivatives on PfUDG activity were investigated. Inhibition of parasite growth was determined *in vitro* using SYBR Green I and compared with results from human cytotoxicity tests.

**Results:**

The native PfUDG was partially purified with a specific activity of 1,811.7 units/mg (113.2 fold purification). After cloning of 966-bp PCR product, the 40-kDa hexa-histidine tagged PfUDG was expressed and identified. The amino acid sequence of PfUDG showed only 24.8% similarity compared with the human enzyme. The biochemical characteristics of PfUDGs were quite similar. They were inhibited by uracil glycosylase inhibitor protein as found in other organisms. Interestingly, recombinant PfUDG was inhibited by two uracil-derived compounds; 1-methoxyethyl-6-(*p*-n-octylanilino)uracil (IC_50_ of 16.75 μM) and 6-(phenylhydrazino)uracil (IC_50_ of 77.5 μM). Both compounds also inhibited parasite growth with IC_50_s of 15.6 and 12.8 μM, respectively. Moreover, 1-methoxyethyl-6-(*p*-n-octylanilino)uracil was not toxic to HepG2 cells, with IC_50_ of > 160 μM while 6-(phenylhydrazino)uracil exhibited cytoxicity, with IC_50_ of 27.5 μM.

**Conclusions:**

The recombinant PfUDG was expressed, characterized and compared to partially purified native PfUDG. Their characteristics were not significantly different. PfUDG differs from human enzyme in its size and predicted amino acid sequence. Two uracil derivatives inhibited PfUDG and parasite growth; however, only one non-cytotoxic compound was found. Therefore, this selective compound can act as a lead compound for anti-malarial development in the future.

## Background

Malaria is an important infectious disease caused by *Plasmodium* parasites, for which *Plasmodium falciparum* is found prevalently and causes virulent symptoms. The disease is endemic in more than 100 countries and causes approximately 655,000 deaths worldwide [1]. The most serious problem for malaria treatment is the development of resistance of the parasites to existing anti-malarial drugs including the most effective drug, artemisinin, which has been used as first-line treatment in many countries [1-6].

The discovery of new drug targets would be an effective strategy for combating multidrug resistant malaria. Many enzymes involved in biosynthesis and metabolic processes such as membrane biosynthesis [7,8], membrane transport [9], proteases [10-12], redox system [13,14] and mitochondrial system [15,16] have been investigated for their potential as anti-malarial drug targets. However, very little is known about malarial DNA repair system and its significance.

DNA repair is essential for parasite existence to prevent gene mutations by both spontaneous mutation during DNA replication of cell division and drug-induced mutation. Base excision repair (BER), for example, plays a major role in removing a damaged base followed by replacement of the correct base into DNA [17,18]. Therefore, enzymes involved in BER should be explored for their roles as new drug targets against malaria. DNA glycosylases are the first enzymes in the BER pathway, and recognizing specific damaged bases, they act as the key enzymes to drive the BER process. Uracil-DNA glycosylase (UDG) is a specific DNA glycosylase for uracil cleavage. This base is introduced by cytosine deamination, which occurs as frequently as 100–500 times/cell/day [19]; therefore, this enzyme plays very important role in preventing G:C to A:T transition mutation.

UDGs of many organisms have been characterized for their biochemical properties, for example the bacterial enzymes (*Escherichia coli*[20,21]), viral enzymes (poxvirus [22], HSV-1 [23]), parasite enzyme (*Trypanosoma cruzi*[24]), and human enzyme [25-27]. Moreover, UDG inhibitors should be considered for targeting to PfUDG. Two types of UDG inhibitors, uracil glycosylase inhibitor (UGI) protein and uracil-derived compounds have been studied. The UGI is a well-known UDG inhibitor isolated from bacteriophage PBS 1 and 2 specific to *Bacillus subtilis*. This protein inhibited UDG of several organisms [28-31]. The uracil-derived compounds, 6-(*p*-alkylanilino)uracils, were reported to inhibit HSV-1 UDG by acting as uracil analogs to bind competitively to the catalytic amino acid residues in the active site motif of UDG [23,32,33].

Because UDG is essential for conservation of genome integrity and normal function of encoding proteins of organisms including the malaria parasite, *P. falciparum*, therefore in this study *P. falciparum* UDG (PfUDG) was successfully cloned and expressed, and the native enzyme was also partially purified. PfUDG activity was determined, and properties of both recombinant and native enzymes were characterized. Inhibitory effects of 12 uracil-derived compounds on enzyme and parasite growth were investigated, and their cytotoxicities were determined. Results obtained were promising for further design of a new anti-malarial drug against *P. falciparum*, especially against drug resistant strains worldwide.

## Methods

### Preparation of partially purified native PfUDG

*Plasmodium falciparum* K1 strain, a chloroquine and pyrimethamine resistant strain from Thailand [34], was cultured in RPMI 1640 (Invitrogen, CA, USA) supplemented with 10% human serum and human red blood cells (RBC) at 37°C in an atmosphere containing 5% CO_2_. The RBC-packed parasites with 20-25% parasitaemia were harvested by centrifugation at 664*xg* at 25°C for 7 min. The parasite pellet was prepared by incubation of RBC-packed parasites with an equal volume of phosphate buffered saline (PBS), pH 7.6, containing 0.15% (w/v) saponin at 37°C for 20 min. The suspension was washed with PBS by centrifugation at 664*xg* at 25°C for 10 min until the supernatant was clear, and the parasite pellet was kept at -80°C until used.

Approximately 2 ml of parasite pellet was resuspended in three volumes of extraction buffer containing 50 mM Tris–HCl pH 7.6, 1 mM EDTA, 2 mM DTT, 1 mM PMSF, and 0.01% NP-40. Parasite cells in suspension were disrupted by Dounce homogenization, and nucleoprotein was extracted by 0.5 M KCl with stirring on ice for 30 min. The suspension was centrifuged at 17,600*xg* for 40 min at 4°C, and the supernatant was dialysed at 4°C overnight against buffer A containing 25 mM Tris–HCl pH 9.0, 1 mM EDTA, 2 mM DTT, 1 mM PMSF, 0.01% NP-40, 5% sucrose, and 20% glycerol.

### Partial purification of native PfUDG

The dialysed crude extract was loaded onto a 1 ml HiTrap™Capto™Q (GE Healthcare Bio-Sciences AB, Uppsala, Sweden) column, and purified by using FPLC®System (Pharmacia Biotech AB, Uppsala, Sweden) at a flow rate of 1 ml/min at 4°C. The unbound fractions were collected, and the column was washed with 10 column volumes (CV) of buffer A. The bound proteins were then eluted with 15 CV of 0-100% KCl linear gradient in buffer A. Both unbound and bound fractions were assayed for UDG enzymatic activity. The fractions containing PfUDG were pooled and dialysed at 4°C overnight against buffer B containing 50 mM HEPES pH 8.5, 1 mM EDTA, 2 mM DTT, 1 mM PMSF, 0.01% NP-40, 5% sucrose, and 20% glycerol. The dialysed fraction was then loaded onto a 1 ml Hitrap™ Heparin HP (GE Healthcare Bio-Sciences AB) column at a flow rate of 0.5 ml/min at 4°C. The unbound proteins were collected and the column was washed with 10 CV of buffer B. The column was then eluted with 15 CV of 0-100% KCl linear gradient in buffer B. Fractions containing PfUDG were pooled and termed native PfUDG.

### Construction of *PfUDG* expression vector

The genomic DNA *P. falciparum* K1 strain was the source of genomic DNA used as a template in PCR to generate full-length *PfUDG*. The full-length gene was amplified by using specific primers: *PfUDG*-forward 5'-CACCATGAATAATCCAACAATT-3' and *PfUDG*-reverse 5'-TTGGGGTAGCTCCCATTTG-3'. The amplification was done by initial denaturing at 95°C for 5 min followed by 35 cycles of reaction with denaturing at 95°C for 1 min, annealing at 58°C for 1 min, extension at 72.5°C for 1 min after each cycle, and final extension at 72.5°C for 3 min. The PCR product was analysed by 1% agarose gel electrophoresis. The amplified full-length *PfUDG* was cloned into pET101/D-TOPO expression vector using Champion™pET Directional TOPO Expression System (Invitrogen) at a 1.5 to 1 molar ratio of the gene fragment and the vector. The constructed vector was named pET101D-PfUDG, and the sequence was analysed for base sequence correction by BioDesign, Pathumthani, Thailand.

### Expression and purification of recombinant PfUDG

The pET101D-PfUDG vector was transformed into *E. coli* Rosetta (DE3) pLysS and grown on LB agar containing 100 μg/ml ampicillin and 20 μg/ml chloramphenicol at 37°C overnight. A single colony was picked and grown in 10 ml of LB broth containing 100 μg/ml ampicillin and 20 μg/ml chloramphenicol at 37°C overnight. The overnight culture was inoculated into 1 L of LB broth containing 0.5 M sorbitol, 100 μg/ml ampicillin and 20 μg/ml chloramphenicol, and incubated at 37°C with shaking at 180 rpm until the optical density at 600 nm reached 0.4. PfUDG expression was induced with 0.5 mM isopropyl β-D-1-thiogalactopyranoside (IPTG), and bacteria were further incubated at 37°C with shaking for 1 hr. The bacteria were then harvested by centrifugation at 1,600*xg* for 20 min, washed with buffer, pH 8.0, containing 50 mM NaH_2_PO_4_ and 300 mM NaCl, and centrifuged at 1,600*xg* for 20 min. The bacterial pellet was resuspended in 200 ml of cold lysis buffer, pH 8.0, containing 50 mM NaH_2_PO_4_, 300 mM NaCl, 10 mM imidazole, and 1 mM PMSF, and disrupted by a XL 2020 Sonicator® Ultrasonic Processor XL (Heat System Inc., NY, USA). The total soluble protein was fractionated by centrifugation at 17,600*xg* at 4°C for 20 min. The expressed protein was analysed by SDS-PAGE.

The total soluble protein was loaded onto a Ni-NTA agarose affinity column (QIAGEN, Hilden, Germany) pre-equilibrated with 10 CV of cold lysis buffer with gravity flow. The column was washed continuously with 12 CV of cold washing buffer, pH 8.0, containing 50 mM NaH_2_PO_4_, 300 mM NaCl, 20 mM imidazole, and 1 mM PMSF, and protein was then eluted with 8 CV of cold eluting buffer, pH 8.0, containing 50 mM NaH_2_PO_4_, 300 mM NaCl, 250 mM imidazole, and 1 mM PMSF. The flowthrough, washed and eluted fractions were collected, and protein concentration was measured by Bradford’s assay (Bio-Rad Laboratories, CA, USA). The protein purity was analysed using SDS-PAGE.

### Identification of PfUDG by LC-MS/MS and 3D modelling of PfUDG

The expected protein bands were cut from the gel and digested with trypsin to get peptide fragments. The pattern of peptide fragments and amino acid sequences were analysed using LC-MS/MS and identified by MASCOT software [35]. The 3D structure of PfUDG was built from predicted amino acid sequences of the full-length PfUDG protein compared to human UDG PDB code 3FCF using SWISS MODEL modelling server in http://swissmodel.expasy.org[36-38]. Geometry optimization was performed in a water box plus ions, and geometry of conformers was validated by PROCHECK [39].

### Preparation of UDG activity assay substrates

The 41-mer uracil-containing single stranded oligonucleotide (oligo-U) and various 41-mer uracil-containing double stranded oligonucleotides, A:U, T:U, C:U, and G:U, were used as substrates in PfUDG activity assay. The sequences of oligonucleotides are shown in Table 1.

**Table 1:** Composition of single stranded oligonucleotides used in UDG activity assay

**Oligonucleotide**	**Sequence: 5'-3'**
oligo-U	GAC ACG TCA GAT AGC ATG ACA **U**CG AGC TGC AGG ACT GAT CT
oligo-A	AGA TCA GTC CTG CAG CTC G**A**T GTC ATG CTA TCT GAC GTG TC
oligo-T	AGA TCA GTC CTG CAG CTC G**T**T GTC ATG CTA TCT GAC GTG TC
oligo-G	AGA TCA GTC CTG CAG CTC G**G**T GTC ATG CTA TCT GAC GTG TC
oligo-C	AGA TCA GTC CTG CAG CTC G**C**T GTC ATG CTA TCT GAC GTG TC

The oligo-U was 5' end radiolabelled with γ-^32^P ATP. A 100 μl reaction volume containing 70 mM Tris–HCl pH 7.6, 10 mM MgCl_2_, 5 mM DTT, 1 unit T4 polynucleotide kinase (New England BioLabs, MA, USA), 20 pmol γ-^32^P ATP (Perkin Elmer, MA, USA) and 200 pmol oligo-U, was incubated at 37°C for 30 min. The reaction was terminated by heating the mixture at 65°C for 20 min. The double stranded substrates were prepared by annealing 100 pmol of ^32^P-labelled single stranded oligo-U to single stranded oligo-A, -T, -C, and -G in 50 μl reaction volumes containing 70 mM Tris–HCl pH 7.6, 10 mM MgCl_2_, and 5 mM DTT. The reactions were initially heated at 95°C for 5 min and allowed to cool to room temperature for 3 hr.

### Enzymatic assay and biochemical characterization

The UDG activity assay (20 μl) consisted of standard reaction buffer containing 20 mM Tris–HCl pH 8.0, 1 mM DTT, 1 mM EDTA, 50 mM NaCl, 100 μg/ml BSA, 10 pmol ^32^P-labelled oligo-U, and 20 ng of PfUDG at 37°C for 20 min. The glycosylase activity was stopped by heating at 100°C for 1 min, the resulting apurinic/apyrimidinic site (AP site) was incised by hot alkaline treatment with 0.1 M NaOH (final concentration) at 95°C for 10 min, and the reaction was neutralized with 0.1 M HCl (final concentration). The AP lyase activity assay of PfUDG was examined under the same condition as UDG activity assay except without hot alkaline treatment. Reaction products were analysed on 8 M urea-16% denaturing PAGE and exposed to X-ray film for autoradiography for visualization. The radioactive intensity of sliced substrate and product bands from the gel were measured in a 1450 MicroBeta® Trilux Liquid Scintillation Counter (Perkin Elmer) as counts per minute (cpm). The amounts of substrate and product (pmol) were determined from radioactive intensity. One unit of activity is defined as the amount of PfUDG required for releasing 1 pmol of uracil in 1 min at 37°C in standard reaction buffer (20 mM Tris–HCl pH 8.0, 50 mM NaCl, 1 mM EDTA, 1 mM DTT, and 100 μg/ml BSA) with ^32^P-labelled single stranded oligo-U substrate.

Biochemical properties of native and recombinant PfUDGs were characterized based on UDG activity under different reaction conditions including temperature, pH, salt concentration, cofactors, and substrate preference. The effects of various temperatures ranging from 4-65°C on enzyme activity were tested. PfUDG was also tested at different buffer conditions: phosphate-citrate buffer for pH 4–7, 20 mM Tris–HCl for pH 7.5-8.5, and glycine-NaOH buffer for pH 9–10.5. Effects of NaCl on PfUDG were investigated by using various NaCl concentrations (5–500 mM). In addition, various concentrations of divalent cation cofactors including MgCl_2_, MnCl_2_, and CaCl_2_ at 1, 10, 50, and 100 mM were tested. Relative activity of PfUDG was determined as above by radioactive intensity (cpm) of product divided by total cpm of substrate and product.

To determine steady state kinetic parameters (*K*_m_, V_max_) for different substrates of the recombinant PfUDG, concentrations of ^32^P-labelled substrates were varied from 0.01 to 3.5 μM. The reaction was initiated by adding 2.5 nM PfUDG (2 ng) to the reaction mixture and incubated at 37°C for 10 min. The kinetic parameters, *K*_m_ and *k*_cat_, were calculated based on the Michaelis-Menten equation using non-linear least square algorithm in SigmaPlot 12.0.

### Inhibitory effects of UDG inhibitors on PfUDG

Two types of UDG inhibitors, UGI protein and synthetic uracil-derived compounds, were tested for their inhibitory effects on PfUDG. UGI protein of *B. subtilis* bacteriophage PBS1 (New England BioLabs) was used against native and recombinant PfUDG with 10 pmol ^32^P-labelled single stranded oligo-U in the standard reaction buffer at 37°C for 20 min. The synthetic uracil-derived compounds were also used at various concentrations (5–640 μM) with 20 ng of recombinant PfUDG and 10 pmol ^32^P-labelled single stranded oligo-U in the standard reaction buffer at 37°C for 20 min. The same 0.4% (v/v final concentration) of DMSO was used as solvent control to determine its effects on enzyme activity. The dose–response curves of relative activities of PfUDG and various compound concentrations were generated, and half-maximal inhibitory concentration (IC_50_), the concentration of compound to inhibit enzyme activity by 50%, was obtained using SigmaPlot 12.0.

### Inhibition of parasite growth using an *in vitro* assay

The synchronized ring stage of *P. falciparum* K1 strain was prepared from mixed stage parasite culture by 5% D-sorbitol treatment. The ring stage parasite was then mixed with culture medium containing RPMI 1640 supplemented with 10% human serum. Various concentrations of each uracil-derived compound were tested at 2-fold serial dilutions in triplicate. Their inhibitory effects on parasite growth were determined by SYBR Green I based assay [40]. The fluorescent intensity was measured by Varioskan Flash Multimode Reader (Thermo Scientific, Vantaa, Finland) with excitation and emission wavelengths of 485 and 535 nm, respectively. The percentage of fluorescent intensity at each compound concentration compared with negative control was indicative of the parasite growth. The dose–response curves of fluorescent intensity and compound concentrations were generated, and IC_50_, the concentration of compound to inhibit parasite growth by 50%, was obtained using SigmaPlot 12.0.

### Cytotoxicity test of active uracil-derived compounds

The uracil-derived compounds showing inhibitory effects on PfUDG were assayed for cytotoxicity to human Caucasian hepatocyte carcinoma cell line ATCC HB 8665 (HepG2) by *in vitro* cytotoxicity test [41]. The methylthiazol tetrazolium (MTT)-dye based colorimetric microtitration assay was used for detecting cell survival based on the metabolism of tetrazolium to formazan by viable cells. The OD of formazan was measured by a Molecular Devices Microplate Reader (Molecular Devices, CA, USA) at a wavelength of 570 nm. The OD_570_ represented the cell survival, and the percentage of cell survival at each compound concentration was determined by comparing with the OD_570_ of negative control lacking compounds. The dose–response curves of % cell survival, and compound concentrations and IC_50_ were obtained. IC_50_ representing the concentration of compounds providing 50% cell survival indicated the cytotoxicity of compounds.

## Results

### Partial purification of PfUDG from parasite crude extract

**Figure 1:**
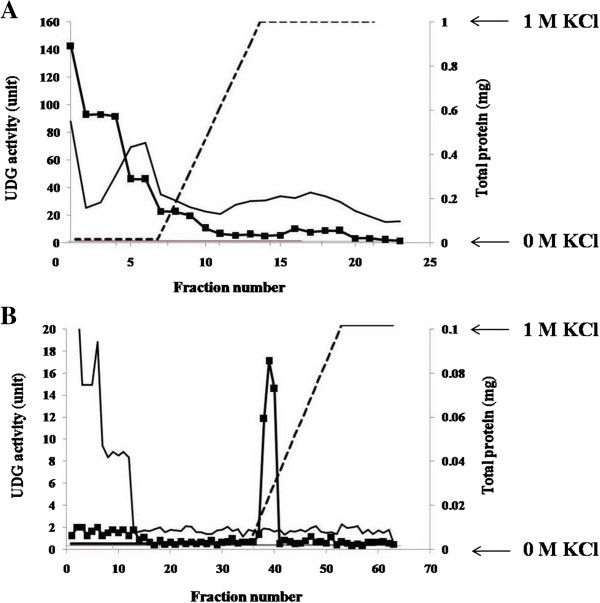
**Partial purification of native PfUDG from *P. falciparum *crude extract. A**, parasite crude extract was purified on the Capto™Q column with 0-100% KCl linear gradient; **B**, Capto™Q pooled fraction containing PfUDG was purified on the HiTrap™ Heparin HP with 0-100% linear KCl gradient, ■; UDG activity, —; total protein, ---; % KCl.

The presence of annotated *PfUDG* in the *Plasmodium* genome suggests that *P. falciparum* would have DNA repair systems similar to those found in other organisms [21-26]. To address its existence and activity in *P. falciparum*, the native PfUDG was partially purified from parasite extract using anion exchange and Heparin Sepharose chromatography. The results of the partial purification of PfUDG are summarized in Table 2. PfUDG activity was found in unbound fractions for anion exchange column eluted with 0.1-0.3 M KCl for Heparin Sepharose column (Figure 1). From 2 ml of parasite pellet, 24 μg of 113 fold-purified PfUDG with a specific activity of 1,811.7 units/mg protein and 2.91% yield were recovered. The result indicates that PfUDG is functionally active during the parasite development. Due to the limit enzyme amount obtained, for further study, the heterologous expression of PfUDG was persuaded. Nonetheless, some biochemical properties of this partially purified native protein were compared to those of the recombinant PfUDG, to determine if the latter can be used as a representative of its native enzyme.

**Table 2:** Partial purification of native PfUDG

**Fraction**	**Volume (ml)**	**Total protein (mg)**	**Total activity (unit)**	**Specific activity**	**Yield (%)**	**Purification fold**
Crude extract	18	93.24	1,492.65	16	100	-
Capto™Q (unbound)	6.7	3.64	705.24	193.7	47.25	12.1
HiTrap™ Heparin	0.6	0.024	43.48	1,811.7	2.91	113.2

### Nucleotide and amino acid sequences analysis of PfUDG

The 966-bp full-length *PfUDG* of *P. falciparum* strain K1 was constructed into pET101/D-TOPO expression vector. The nucleotide sequences showed 99.7% similarity compared to chloroquine and pyrimethamine-sensitive *P. falciparum* 3D7 [NCBI nucleotide reference sequence: XM_001348285.1] since a single base change (C→A) was found at the position 469. The base change resulted in threonine of PfUDG K1 instead of proline of its counterpart in strain 3D7. In addition, only 24.8% amino acid sequence similarity of PfUDG was found compared with the human enzyme. Moreover, the predicted amino acid sequences of PfUDG K1 were much different from UDGs of other organisms except for *Plasmodium vivax* as demonstrated in Table 3. The predicted molecular mass of PfUDG derived from amino acid sequences was about 37.461 kDa [PlasmoDB: PF3D7_1415000].

**Table 3:** Amino acid sequence similarity of PfUDG K1 compared with UDGs from other organisms

**Organisms**	**Accession numbers of NCBI protein reference sequence**	**Similarity (%)**
*P. falciparum* 3D7	XP_001348321.1	99.7
*P. falciparum* IT	PF IT_1416000*	99.7
*Plasmodium vivax*	XP_001616803.1	61.2
*Homo sapiens* (hUNG2)	NP_550433.1	24.8
*Homo sapiens* (hUNG1)	NP_003353.1	24.8
*E. coli* K12	NP_417075.1	31.0
*Saccharomyces cerevisiae* S288	NP_013691.1	26.1
*Mus musculus* isoform A	NP_001035781.1	28.6
*Mus musculus* isoform B	NP_035807.2	27.8
*T. cruzi* CL Brener	XP_821835.1	32.1
Herpes simplex virus type 1	NP_044603.1	24.8
Herpes simplex virus type 2	NP_044471.2	24.8

*Gene ID (available in PlasmoDB.org).

### Expression and purification of recombinant PfUDG

**Figure 2:**
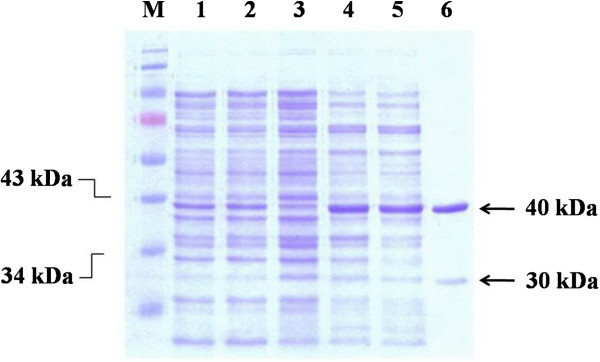
**Analysis of recombinant PfUDG expression by 12**% **SDS**-**PAGE.** The gel was stained with Coomassie blue R250. Lane M, molecular weight markers; lane 1, non-induced whole cells of *E. coli* Rosetta expression host; lane 2, 0.5 mM IPTG-induced whole cells of *E. coli* Rosetta expression host; lane 3, non-induced whole cell of *E. coli* Rosetta carrying *PfUDG*-constructed vector; lane 4, 0.5 mM IPTG-induced whole cell of *E. coli* Rosetta carrying *PfUDG*-constructed vector; lane 5, total soluble protein of 0.5 mM IPTG-induced whole cell of *E. coli* Rosetta carrying *PfUDG*-constructed vector; lane 6, Ni-NTA agarose purified protein. The arrows indicate the completed (40 and 30 kDa) 6-residue histidine-tagged PfUDG.

After 1 L scale expresssion, 3.75 g of bacteria were obtained. Approximately 190 mg of total soluble protein were obtained after disruption, and the recombinant PfUDG was further purified by Ni-NTA agarose column. After purification, two protein bands, ~40 and ~30 kDa, were observed (Figure 2) with the total amount of 9 mg. The 40-kDa protein size agrees with the calculated molecular mass for the sum of 37 kDa PfUDG and 2.4 kDa of 20 amino acids of C-terminal sequence containing 6 histidine and V5 epitope.

### Identification of PfUDG by LC-MS/MS and 3D modelling of PfUDG

**Figure 3:**
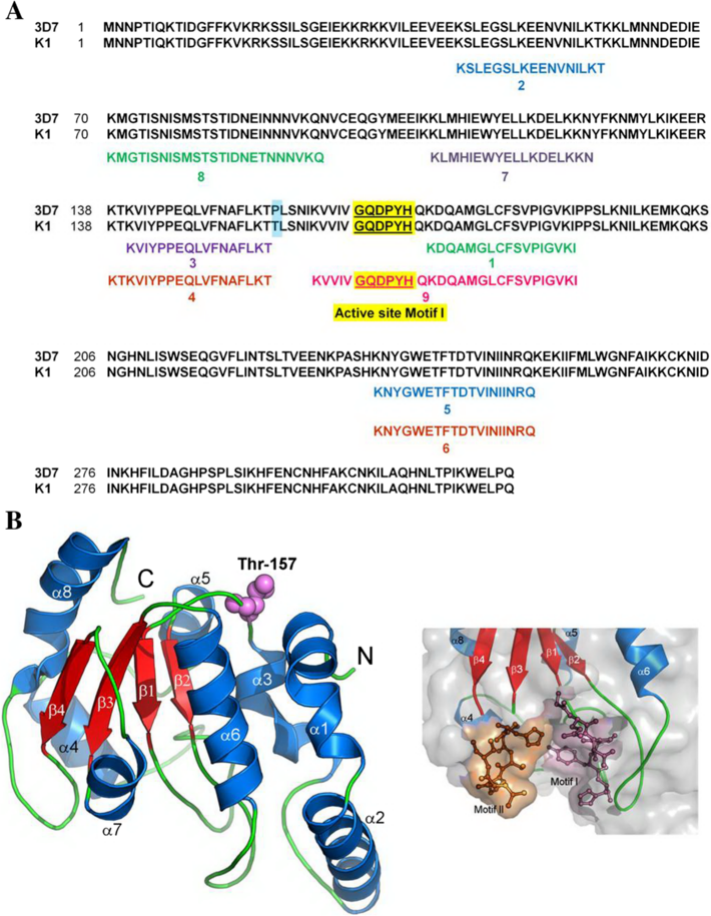
**Amino acid sequences and 3D secondary structure of PfUDG. A**, Alignments of 9 peptide fragments of PfUDG K1 from LC-MS/MS to complete amino acid sequences of PfUDG 3D7 and K1. Blue box, the different amino acid between PfUDG 3D7 and K1; yellow box, conserved amino acid sequences of active site motif I found in fragment 9; **B**-left, 3D model of PfUDG K1. Cartoon model shows α-helices, blue, β-sheets, red, coils/loops, green. Thr157 is represented as spheres. N, N terminus and C, C terminus. B-right, Molecular surface represents two motifs of the enzyme active sites and their residues (bonds and sticks).

The identities of 40- and 30-kDa proteins were analysed using tryptic digestion in association with LC-MS/MS. The peptide fragmentation and mass spectrometric analysis showed that nine peptide fragments of both proteins were matched to *P. falciparum* 3D7 UDG sequence with scores of 278 and 177 respectively (Figure 3A), for which one of these peptide fragments contained the sequence of active site Motif I (water activating motif, GQDPYH). Therefore, the 40-kDa protein (80% of total purified protein) was suggested to be a full-length PfUDG while the 30-kDa protein (20% of total purified protein) was a truncated/degraded form.

The 3D structure was built from the 214 amino acid sequence of PfUDG K1 using human UDG as a template (Figure 3B). This structure presented 8 α helices and 4 paralled β sheets. The active site motif I (Gly167, Gln168, Asp169, Pro170, Tyr171, and His172) was located at a loop connecting β1 strand and α4 and motif II was located at the loop linking β4 strand and α8 near the C terminus of the enzyme. A single amino acid different at Pro157 for *P. falciparum* strain 3D7 compared to Thr157 of *P. falciparum* strain K1 is located at the loop connecting the α3 and β1 strands. Stereochemical quality of the PfUDG structure generated was validated by PROCHECK and presented as Ramachandran Plot determined from degree of Phi and Psi angles of each amino acid residue. The result demonstrated 90.2% amino acid residues in the most favoured region, 8.2% for additional allowed region, 1.5% for generously allowed region, and 0.0% for disallowed region. Moreover, the root mean square deviation of PfUDG K1 model was 1.18 compared to the template analysed by Swiss-PdbViewer Software suggesting the appropriate structure of the PfUDG modelled.

### Enzymatic activity and biochemical characterization

**Figure 4:**
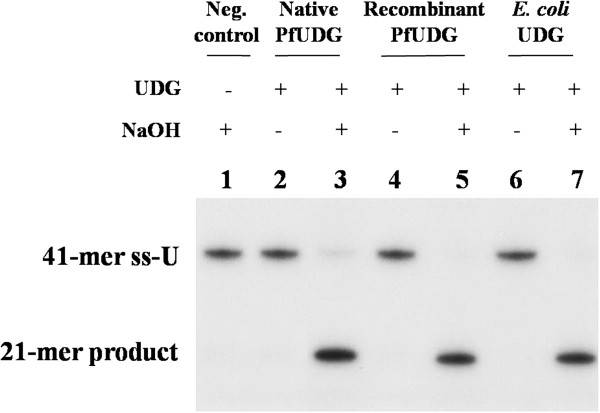
**Enzymatic activity of native and recombinant PfUDGs with**^**32**^**P**-**labelled single stranded oligo**-**U.** Lane 1, the negative control reaction lacking PfUDGs; lanes 2, 4, and 6, the reactions without hot alkaline treatment (AP lyase activity assay) were performed by native , recombinant PfUDG and *E. coli* UDG respectively; lanes 3, 5, and 7, the reactions with hot alkaline treatment (glycosylase activity assay) were performed by native and recombinant PfUDG and *E. coli* UDG respectively.

The glycosylase activity of both native and recombinant PfUDGs was determined using 5' end radiolabelled 41-mer oligonucleotide, for which uracil is present in the middle of the sequence. Upon the base excision by UDG and hot alkaline treatment, the 21-mer product would be generated. Both native and recombinant PfUDGs possessed UDG activity but lacked of the AP lyase activity because there was no product generated without hot alkaline treatment (Figure 4). Therefore, PfUDG was a monofunctional enzyme.

**Figure 5:**
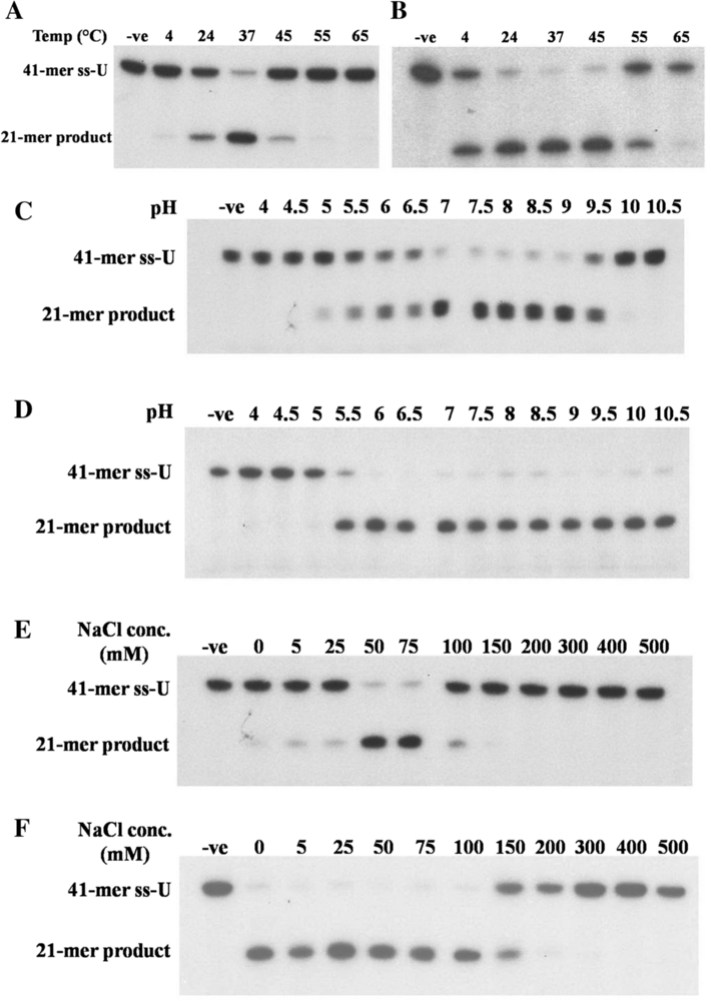
**Effects of various temperatures, pH and salt concentrations on native and recombinant PfUDGs.** Both native **(A, C and E)** and recombinant **(B, D and F)** PfUDG activities were investigated under various conditions of temperature, pH and NaCl concentration. The negative control reaction without PfUDG is designated as –ve.

The effects of temperature on PfUDG were examined from 4 to 65°C. Both native and recombinant PfUDGs showed highest relative activity at 37°C with 80% and 93%, respectively compared to the initial substrate (100%) (Figure 5A, 5B). The enzymatic activities decreased at either lower or higher temperatures than 37°C. For the pH optimum, the results demonstrated that the native enzyme functioned in the narrow range of pH (7–9) compared to that of the recombinant enzyme (6–10.5) (Figure 5C, 5D). However, both enzymes worked well under alkaline conditions. The optimal salt concentration for the native enzyme was 50–75 mM NaCl with 82% relative activity, whereas enzyme activity was not observed at lower NaCl concentrations (Figure 5E, 5F). However, more than 50% inhibition was found at 100 mM NaCl, and the higher salt concentrations (150–500 mM) showed complete inhibition. High activity of the recombinant enzyme (87-94%) was observed at 0–100 mM NaCl, while more than 50% enzyme inhibition was seen at 150 mM and complete inhibition occurred at 200–500 mM NaCl. However, the optimal salt concentrations were 50–75 mM which is in the same range as found for the native enzyme.

**Figure 6:**
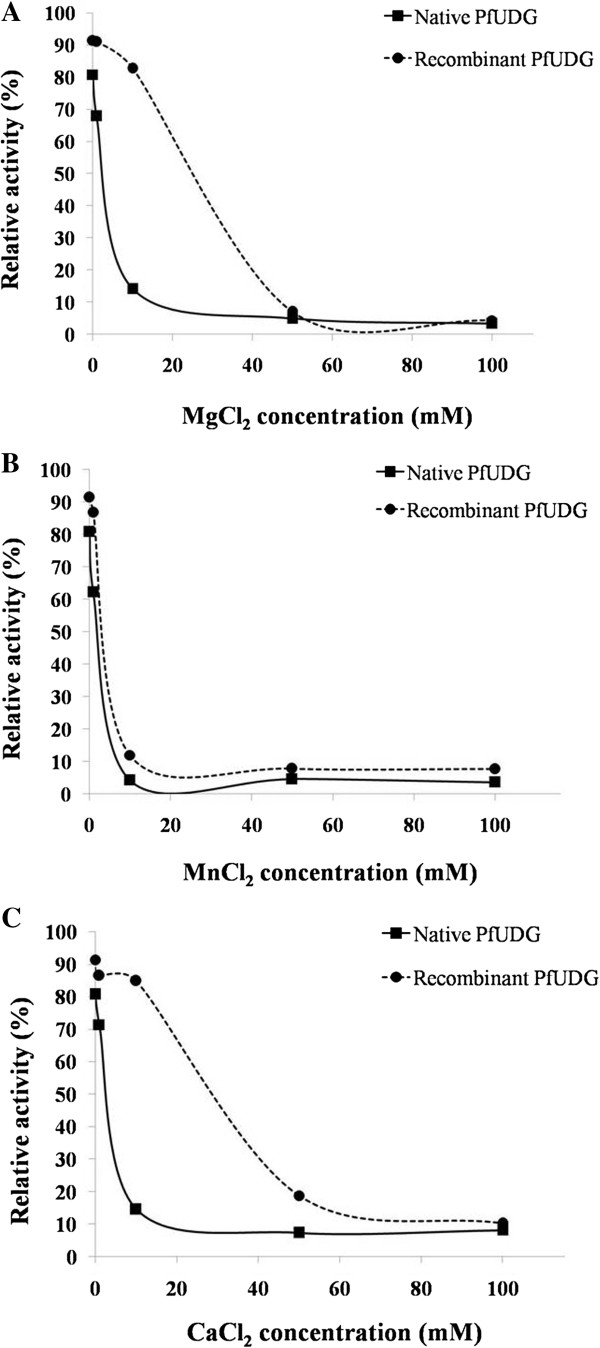
**Effects of divalent cation cofactors on native and recombinant PfUDGs.** PfUDG activities of native and recombinant enzymes were investigated under the assayed condition with the presence of 1, 10, 50 and 100 mM of MgCl_2_**(A)**, MnCl_2_**(B)** and CaCl_2_**(C)**.

**Figure 7:**
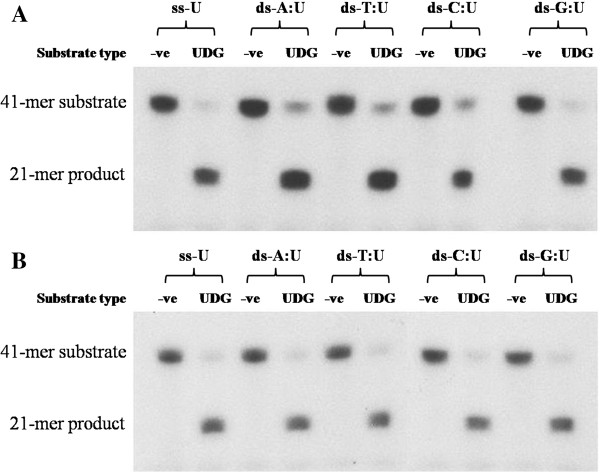
**Effects of substrate types on native and recombinant PfUDGs.** Native **(A)** and recombinant **(B)** PfUDG activities were investigated by using different substrates. ss-U, single stranded oligo-U; ds-A:U, double stranded A:U; ds-T:U, double stranded T:U; ds-C:U, double stranded C:U; ds-G:U, double stranded G:U. The negative control reaction without PfUDG is designated as -ve.

The results indicated that the catalytic reaction of these enzymes did not require any cofactors. In addition, both native and recombinant PfUDGs were inhibited by increasing concentrations of cofactors (Figure 6). For the substrate preference, both PfUDG enzymes were able to remove uracil from single stranded oligo-U (ss-U) and double stranded (ds) A:U, T:U, C:U, and G:U with similar relative activities (Figure 7).

Based on kinetic parameters of recombinant PfUDG, there were no significant differences of any kinetic constants using ss-U and ds-A:U as substrates (Table 4). Compared with the kinetic parameters of human nuclear uracil *N*-glycosylase (hUNG2) [27], the substrate affinity (*K*_m_) of PfUDG to ds-A:U was higher than that of hUNG2, while *K*_m_ of PfUDG to ss-U was lower than that of hUNG2. The turnover numbers (*k*_cat_) of hUNG2 were about 2- and 40-fold higher than those of PfUDG both in ds-A:U and ss-U. The specificity constants (*k*_cat_/*K*_m_) of hUNG2 for ds-A:U and ss-U were about 5- and 30-fold higher than those of PfUDG.

**Table 4:** Kinetic parameters of recombinant PfUDG

**Substrate**	***K***_**m**_**(μM)**		***k***_**cat**_**(min**^-**1**^**)**		***k***_**cat**_/***K***_**m**_**(min**^-**1**^**/μM**^-**1**^**)**	
	**PfUDG**	hUNG2*	**PfUDG**	hUNG2*	**PfUDG**	hUNG2*
ss-U	1.633	2.2 ± 0.2	59.6	2,768 ± 105	36.50	1,258
ds-A:U	1.656	0.7 ± 0.1	60.0	137 ± 4	36.23	187

Each reaction was performed in 2 independent experiments in a standard reaction buffer containing 20 mM Tris–HCl, pH 8.0, 1 mM DTT, 1 mM EDTA, 50 mM NaCl, 100 μg/ml BSA, 2.5 nM recombinant PfUDG, and 0.01-3.5 μM ^32^P-labelled single strand oligo-U (ss-U) or double stranded A:U (ds-A:U) at 37°C for 10 min.

*[27].

### Inhibitory effects of inhibitors on PfUDG activity, parasite growth and their cytotoxicity

**Figure 8:**
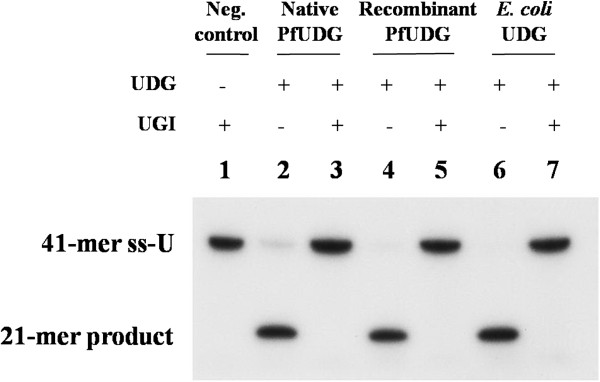
**Inhibitory effects of UGI of *B. subtilis *bacteriophage PBS1 on native and recombinant PfUDGs.** Lane 1, the negative control lacking PfUDG; lanes 2, 4, and 6, the reactions were performed with native and recombinant PfUDG and *E. coli* UDG, respectively; lanes 3, 5, and 7, the reactions were performed with UGI against native and recombinant PfUDG and *E. coli* UDG, respectively.

The inhibitory effect of UGI protein of *B. subtilis* bacteriophage PBS1 on native and recombinant PfUDGs was analysed using 1:1 molar ratio of PfUDG and UGI. Both native and recombinant PfUDGs were completely inhibited by UGI (Figure 8). After testing of 12 uracil-derived compounds against recombinant PfUDG, only two of them, namely 1-methoxyethyl-6-(*p*-n-octylanilino)uracil and 6-(phenylhydrazino)uracil, showed inhibitory effects on PfUDG activity with IC_50_s of 16.75 and 77.5 μM, respectively. The rest showed no inhibition even at a concentration of 400 μM. However, all compounds showed inhibitory effects on parasite growth with different IC_50_s. 1-Methoxyethyl-6-(*p*-n-octylanilino)uracil and 6-(phenylhydrazino)uracil showed IC_50_s of 15.6 and 12.8 μM, respectively, whereas the most effective compound against parasite growth was 6-(*p*-n-heptylanilino)uracil with IC_50_ of 5.0 μM. Additionally, 1-methoxyethyl-6-(*p*-n-octylanilino)uracil was not toxic to the HepG2 cell line as indicated by its IC_50_ value, which is 10 fold greater than that of the parasite (IC_50_ of >160 μM vs. 15.6 μM) (Table 5).

**Table 5:** **Comparisons of IC**_**50**_**s of uracil**-**derived compounds in their inhibitory effects on recombinant PfUDG activity**, ***P. falciparum *** **growth and cytotoxicity to HepG2 cell lines**

**No**.	**Compound**	**IC**_**50**_**(μM)**		
		**PfUDG**	***P. falciparum***	**HepG2 cytotoxicity**
1	1-Methoxyethyl-6-(*p*-n-octylanilino)uracil	16.75	15.6	> 160
2	6-(Phenylhydrazino)uracil	77.5	12.8	27.5 ± 4.58
3	6-(*p*-n-Heptylanilino)uracil	> 400	5.0	ND
4	6-(4-Methylanilino)uracil	> 400	7.1	ND
5	6-(*p*-n-Propylanilino)uracil	> 400	7.9	ND
6	Uracil	> 400	10.0	ND
7	6-(*p*-n-Butylanilino)uracil	> 400	10.0	ND
8	3-(4-Hydroxybutyl)-6-(3-ethyl-4-methylanilino)uracil	> 400	10.2	ND
9	6-(*p*-n-Pentylanilino)uracil	> 400	10.5	ND
10	6-(*p*-i-Pentylanilino)uracil	> 400	11.0	ND
11	6-(*p*-i-Propylanilino)uracil	> 400	12.6	ND
12	6-(*p*-i-Butylanilino)uracil	> 400	16.5	ND

ND = not-determined.

## Discussion

Usually, UDG works as the key enzyme in the BER pathway to recognize the uracil base occurring abnormally during DNA replication [42,43]. Uracil causing G:C to A:T transition mutations in DNA is harmful to essential genes of all organisms. Because this enzyme acts in the primary step to direct further steps of the DNA repair pathway, inhibition of this enzyme would affect DNA repair of *P. falciparum* and eventually may lead to parasite death.

In this study, the existence and activity of PfUDG was firstly confirmed by isolation and partial purification of the native PfUDG from parasite extracts using anion exchange and Heparin Sepharose chromatography. The late trophozoites and schizonts were used in this experiment, because they are involved in cell division in which incorrect DNA replication should occur frequently; DNA repair is essential for maintenance of genome integrity during DNA replication to prevent mutation. The frequency of generated uracil in parasite DNA should be high during DNA replication similar to other organisms including human [19,43].

Since the calculated pI value of PfUDG is rather high (pI 9.78), finding PfUDG in a flow through fraction when using an anion exchange column with buffer system at pH 9 was expected. This step was efficient as 50% UDG activity was recovered, while a large amount of other proteins was removed (Table 2). After using two columns, we could not further purify PfUDG to near homogeneity due to the low amount of enzyme activity, instability during subsequent purification steps even in the presence of protease inhibitors, and lack of an affinity column for UDG purification. In addition, cultivation of *P. falciparum* was laborious and much more medium, human red blood cells and serum would be required to obtain a higher amount of parasites. Therefore, heterologous expression of PfUDG was an option pursued in this study.

*Escherichia coli* is among the most widely used hosts for heterologous protein production due to its fast growth rate in cheap media, known genetic property, and ease of use and genetic manipulation. However, expression of *Plasmodium* proteins in *E. coli* can sometimes be challenging [44,45]. Expression of PfUDG in a general expression BL21 DE3 host yielded PfUDG mostly as an insoluble protein. However, Rosetta (DE3) pLysS - a BL21 derivative harbouring extra copies of rare tRNAs for codons AUA (Ile), AGG (Arg), AGA (Arg), CUA (Leu), CCC (Pro), and GGA (Gly) significantly improved PfUDG solubility. These rare codons accounted for 53% AUA, 75% AGG/AGA, 7% CUA, 17% CCC, and 50% GGA of total codons encoding each amino acid in PfUDG. With the yield of 9 mg/L culture obtained via the optimized expression and purification protocols, other downstream studies such as biochemical characterization and inhibitor screening could be facilitated. However, a faint band of incomplete protein was observed during protein expression as a ~30-kDa protein (Figure 2). This may be a degraded or truncated form caused by the over-expression of recombinant protein in the *E. coli* host, based on degradation of excess protein by bacterial enzymes. Therefore, bacteria may not be the best expression host for eukaryote PfUDG expression, and the use of a eukaryote expression host should improve the quality and yield of protein expression.

It should be mentioned that sequence comparisons among different *P. falciparum* strains revealed Thr/Pro157 polymorphism. Although Proline is present in other strains, various amino acids including Threonine are observed in other UDGs, suggesting that this polymorphism is unlikely to affect enzyme catalysis. Additionally, this residue is located distantly from the active site based on the PfUDG modelled structure.

Based on a 3D structure of the recombinant PfUDG, PfUDG was similar to other organisms as it contains 8 α-helices and 4-paralled β strands [46]. The two active site motifs are located in positions similar to other UDGs: motif I located at the loop connecting β1 strand and α4 helix, and motif II located at the connecting loop of β4 strand and α8 helix. The stereochemical quality of the PfUDG structure, which was validated by PROCHECK, confirmed that the modelled structure is suitable for study of the molecular interaction between enzyme and inhibitors.

Biochemical properties such as assay condition, substrate preference, and enzyme inhibition by UGI of native and recombinant PfUDGs were characterized. PfUDG is a heat-labile enzyme, and the optimal temperature of both native and recombinant PfUDG was the same at 37°C. This is the physiological temperature of human hosts of parasites. This optimal temperature was similar to that for UDG from other organisms except *T. cruzi* UDG, which showed optimal temperature at 45°C [24]. Both forms of PfUDG worked well in alkaline conditions (pH 7–9), so it is suggested to be one of the pH-dependent enzymes. This result relates to the optimal pH of UDG from other organisms [20,24,27].

The optimal NaCl concentrations for both native and recombinant PfUDGs were 50–75 mM, which are in the same range of UDG from other organisms, for example 50 mM in hUNG2 [27] and 65 mM in *T. cruzi* UDG [24]. The enzyme activity was significantly decreased at higher NaCl concentrations. The high concentrations of neutral salts can produce high ionic strength that inhibits the activity of many enzymes [47].

For the cofactor requirement, the native and recombinant PfUDGs worked effectively without any divalent cation cofactors including Mg^2+^, Mn^2+^ and Ca^2+^. Our results were similar to those for *E. coli* UDG that did not require any cofactors for its function [20,21] while UDG of human [27], vaccinia virus [29] and HSV-2 [48] showed activity with optimal MgCl_2_ at 6 mM, 7.5 mM and 5 mM, respectively. Therefore, PfUDG is suggested as a cofactor-independent enzyme which is significantly different from the human enzyme. High concentrations of divalent cations were suggested to induce structural changes or interact directly with negatively charged groups of the enzyme [49]. The native and recombinant PfUDGs functioned at comparable assay conditions; however, it was noted that the recombinant enzyme showed a broader tolerant range of assay conditions.

Based on the results obtained that PfUDG possesses uracil glycosylase activity for both ss-U and ds-A:U but lacks AP lyase activity, PfUDG belongs to UDG-family I [46]. In human, uracil found in double stranded DNA is generated via cytosine deamination, while that seen in single stranded DNA is due to misincorporation of the nucleotide at the replication fork during DNA replication [27]. Unlike the human enzyme (hUNG2), PfUDG exhibited similar catalytic efficiency (*k*_cat_/*K*_m_) toward ss-U and ds-A:U. The data suggests that PfUDG can serve to remove uracil resulting from deamination damage and misincorporation during DNA replication to prevent gene mutation in the malarial parasite.

Both enzymes were inhibited by UGI protein of bacteriophage PBS1 specific to *B. subtilis* similar to UDG from other organisms [28-31,50]. Therefore, the recombinant enzyme is functionally comparable to the native counterpart and can replace the native enzyme for further studies such as inhibitor screening.

PfUDG was inhibited by two uracil-derived compounds: 1-methoxyethyl-6-(*p*-n-octylanilino)uracil showed a greater inhibitory effect (IC_50_ 16.75 μM) than 6-(phenylhydrazino)uracil (IC_50_ 77.5 μM). Some of the tested compounds were previously studied for their inhibitory effects on HSV-1 UDG purified from HSV-1-infected HeLa cells and purified human HeLa UDG and they inhibited HSV-1 UDG more than human UDG based on their IC_50_s [33]. 6-(4-n-Octylanilino)uracil, an analog of 1-methoxyethyl-6-(*p*-n-octylanilino)uracil) and 6-(4-n-hexylanilino)uracil were used to differentiate HSV-1 and human UDG. Based on our results, four compounds showed no inhibition of PfUDG (IC_50_ of > 400 μM), but demonstrated inhibition of HSV-1 UDG such as 6-(*p*-n-butylanilino)uracil (IC_50_ of 150 μM), 6-(*p*-n-pentylanilino)uracil (IC_50_ of 30 μM), 6-(*p*-i-pentylanilino)uracil (IC_50_ of 140 μM), and 6-(*p*-n-heptylanilino)uracil (IC_50_ of 20 μM) [33]. Therefore, these four compounds can be used to differentiate HSV-1 UDG and PfUDG. This result confirmed that there are some differences between the malarial enzyme (PfUDG) and viral enzyme (HSV-1 UDG). Moreover, 1-methoxyethyl-6-(*p*-n-octylanilino)uracil exhibited significant inhibitory effects on PfUDG (IC_50_ of 16.75 μM) while 6-(4-n-octylanilino)uracil showed no inhibitory effects on human UDG (IC_50_ of > 300 μM) [33]. The 1-methoxyethyl-6-(*p*-n-octylanilino)uracil and other series of alkylanilinouracils should be further tested for their inhibitory effects on human UDG. Among 12 inhibitors, 1-methoxyethyl-6-(*p*-n-octylanilino)uracil exhibited the most potent anti-PfUDG activity with anti-malarial activity. Additionally, this compound was not cytotoxic to HepG2 cells (IC_50_ was 10 folds greater than that of *P. falciparum*). The data here indicate the dissimilar binding sites between the parasite and human enzymes, hence rational design for a selective inhibitor against PfUDG can be achieved. Furthermore, 1-methoxyethyl-6-(*p*-n-octylanilino)uracil can serve as a starting template for development of new antiplasmodial agents. Molecular docking of this analog and PfUDG in comparison to that of human enzyme is being explored to reveal the distinct binding modes and molecular interactions between these enzymes and inhibitor to aid the rational drug design.

## Conclusions

The native and recombinant PfUDGs were characterized for biochemical properties. Results demonstrated that there is no significant difference between them. Therefore, recombinant PfUDG can be used to screen for active compounds targeting this parasite enzyme. The recombinant enzyme was inhibited by two uracil-derived compounds, 1-methoxyethyl-6-(*p*-n-octylanilino)uracil and 6-(phenylhydrazino)uracil, and these compounds inhibited parasite growth. The first compound was not toxic to HepG2 cell lines while the latter one was cytotoxic. These results indicate that PfUDG may act as a good target against malaria, and newly synthesized uracil-derived compounds should be further developed to have the most effective and least toxic anti-malarial drug in the future.

## Competing interests

The authors declare that they have no competing interests.

## Authors’ contributions

All authors contributed in manuscript writing. TS performed most of the laboratory work, data analysis and manuscript preparation. UL participated in designing the molecular work and enzyme kinetics. SM and SS were involved in parasite culture and enzyme purification. UB performed 3D structure analysis and discussion. GEW performed uracil-derived compound synthesis and editing the manuscript. PCP was involved in study design, data analysis, discussion and editing of the manuscript. All authors have already read and approved the manuscript.
